# 1-year quality of life and health-outcomes in patients hospitalised with COVID-19: a longitudinal cohort study

**DOI:** 10.1186/s12931-022-02032-7

**Published:** 2022-05-04

**Authors:** Kate O’Brien, Liam Townsend, Joanne Dowds, Ciarán Bannan, Parthiban Nadarajan, Brian Kent, Niamh Murphy, Gráinne Sheill, Ignacio Martin-Loeches, Emer Guinan

**Affiliations:** 1 Department of Physiotherapy, St. James’s Hospital, Dublin, Ireland; 2 School of Medicine, Trinity College, The University of Dublin, Dublin, Ireland; 3 Department of Infectious Diseases, St. James’s Hospital, Dublin, Ireland; 4 Department of Clinical of Medicine, School of Medicine, Trinity Translational Medicine Institute, Trinity College, The University of Dublin, Dublin, Ireland; 5 Department of Respiratory Medicine, St. James’s Hospital, Dublin, Ireland; 6 Department of Intensive Care Medicine, St. James’s Hospital, Dublin, Ireland; 7 Department of Clinical Medicine, School of Medicine, Trinity College, The University of Dublin, Dublin, Ireland

**Keywords:** COVID-19, Hospitalisation, Physical functioning, Recovery, Fatigue, Breathlessness, Exercise

## Abstract

**Background:**

Published studies suggest physical recovery from the COVID-19 is complex, with many individuals experiencing persistent symptoms. There is a paucity of data investigating the longer-term trajectory of physical recovery from COVID-19.

**Methods:**

A prospective longitudinal design was utilised to investigate the impact COVID-19 has on physical functioning at 10-weeks (T1), 6-months (T2) and 1-year (T3) post-hospital discharge. Objective measures of recovery included 6-Minute Walk Test Distance (6MWTD), frailty (Clinical Frailty Scale), quantification of falls following hospital-discharge, return to work status and exercise levels. Subjective markers included symptoms (COVID-19-Specific Patient Concerns Assessment), fatigue (Chalder Fatigue Score) and health-related quality of life (HrQOL) [Short-Form-36 Health Survey Questionnaire (SF-36-II)]. Univariate analysis was performed using t-test, Wilcoxon rank-sum, and Chi-squared test, paired analysis using one-way analysis of variance and Krustal Wallis testing and correlation analysis with Spearman correlation tests.

**Results:**

Sixty-one subjects participated. Assessments were conducted at a median of 55 days(T1), 242 days(T2), and 430 days(T3) following hospital-discharge. 6MWTD improved significantly overtime (*F* = 10.3, *p* < 0.001) from 365(209)m at T1 to 447(85)m at T3, however remained below population norms and with no associated improvement in perceived exertion. Approximately half (n = 27(51%)) had returned to pre-diagnosis exercise levels at T3. At least one concern/symptom was reported by 74%, 59% and 64% participants at T1, T2 and T3 respectively. Fatigue was the most frequently reported symptom at T1(40%) and T2(49%), while issues with memory/concentration was the most frequently reported at T3(49%). SF-36 scores did not change in any domain over the study period, and scores remained lower than population norms in the domains of physical functioning, energy/vitality, role limitations due to physical problems and general health. Return-to-work rates are low, with 55% of participants returning to work in some capacity, and 31% of participants don’t feel back to full-health at 1-year following infection.

**Conclusion:**

Hospitalised COVID-19 survivors report persistent symptoms, particularly fatigue and breathlessness, low HrQOL scores, sub-optimal exercise levels and continued work absenteeism 1-year following infection, despite some objective recovery of physical functioning. Further research is warranted to explore rehabilitation goals and strategies to optimise patient outcomes during recovery from COVID-19.

**Clinical message:**

Hospitalised COVID-19 survivors report significant ongoing rehabilitation concerns 1-year following infection, despite objective recovery of physical functioning. Our findings suggest those who returned to exercise within 1-year may have less fatigue and breathlessness. The impact of exercise, and other rehabilitative strategies on physical functioning outcomes following COVID-19 should be investigated in future research.

**Supplementary Information:**

The online version contains supplementary material available at 10.1186/s12931-022-02032-7.

## Introduction

In January 2020, the World Health Organisation declared the Severe Acute Respiratory Syndrome Coronavirus 2 (SARS-CoV-2) outbreak a public health emergency. In Ireland, as of the 11th of January 2022, there have been 978,000 confirmed cases of COVID-19 and 5952 associated deaths [1], meaning approximately 19% of the country’s population has contracted the virus. COVID-19 has been established as a severe disease with a substantial burden on global healthcare.

As the number of COVID-19 survivors increases rapidly, there is an urgent need to investigate recovery from the virus, particularly in those who suffered more severe acute illness necessitating hospital admission. Importantly, a growing number of survivors are experiencing persistent symptoms after apparent resolution of viral infection, a condition known as *Long COVID* or *Post-Acute COVID-19 Syndrome (PACS)* [2].

There are multiple studies investigating the short- to medium-term outcomes following COVID-19 infection. Many hospitalised patients show severe impairment in physical functioning and have difficulty carrying out basic activities of daily living (ADLs) at time of hospital-discharge [3]. Functional impairment can persist following discharge, most frequently characterised by dyspnoea on exertion and fatigue [4, 5]. The trajectory of these symptoms during recovery remains understudied. Dyspnoea, fatigue and the other reported symptoms of *Long COVID* or PACS may affect ability to exercise, completion of ADLs, and ability to return to work, thus reducing quality of life. Not only are these persistent symptoms burdensome but they may also carry a sense of stigma [6]. A period of adjustment may follow acute infection, particularly for those who had few co-morbidities and were highly functioning prior to their infection with COVID-19.

Persistent respiratory symptoms, fatigue, sleep difficulties and impaired submaximal exercise capacity have reported up to 8-months after infection [7–11]. While greater severity of initial infection is associated with reduced diffusion capacity for carbon monoxide in pulmonary function tests (PFTs) and persistent radiological abnormalities on high-resolution computed tomography (HRCT) [10, 11], it is becoming more apparent that there may be a discordance between symptom burden and clinical findings [12]. Sixty-seven percent of COVID-19 survivors are symptomatic with breathlessness, fatigue, and cough three-months following infection, despite lack of correlation with pulmonary function tests (PFTs) or the presence of such radiological abnormalities [7], with this discordance persisting up to 6-months following infection [9].

Experience from other Coronavirus outbreaks including Severe Acute Respiratory Syndrome (SARS-CoV-1), suggests that some patients will experience long-term complications including fatigue, depression and anxiety, vocational problems and reduced quality of life up to 12-months following infection [13]. Therefore, it is unsurprising that an increase in anxiety and mood disorders has also been reported 6-months after infection with COVID-19 [14].

To date, research investigating recovery from COVID-19 relies primarily on single time-point assessment and/or have limited follow-up time. There is a clear need to assess the trajectory of recovery from COVID-19 over a longer period.

It is possible that return to previous levels of function will be slow, and for some, not possible. The aim of this study was to prospectively investigate objective physical recovery and self-reported well-being following COVID-19 amongst a heterogeneous group of hospitalised patients at 10-weeks, 6-months and 1-year following hospital-discharge.

## Methods

### Design

A prospective longitudinal design was used to investigate the impact COVID-19 has on objective and subjective markers of physical functioning at 10-weeks (T1), 6-months (T2) and 1-year (T3) post-hospital discharge.

### Study population and recruitment

This study included a consecutively enrolled cohort of patients hospitalised with COVID-19 in St. James’s Hospital, Dublin, Ireland’s largest acute academic teaching hospital. Patients were referred to this clinic at time of discharge, and to the clinic physiotherapy service for assessment of persistent issues with physical functioning on the day of their clinic appointment. All patients who were referred to the clinic physiotherapy service between May and November 2020 were recruited to participate in the study. Recruitment ceased when the physiotherapists could no longer attend clinics due to lack of resources and increasing acute inpatient workloads. Eligibility criteria included hospitalisation for COVID-19 (confirmed by positive SARS-CoV-2 Polymerase Chain Reaction test), ability to complete study questionnaires in English and aged ≥ 18 years. Written informed consent was obtained at T1. Ethical approval was obtained from the Tallaght University Hospital (TUH)/St James’s Hospital (SJH) Joint Research Ethics Committee (REC: 2020–11 List 43—Amendment [15]).

### Measurements

#### Objective measurements

Clinical data pertaining to the acute hospital stay were obtained through electronic chart reviews.

At the initial clinic appointment (T1), two physiotherapists assessed aerobic capacity and endurance using the 6-Minute Walk Test (6MWT) and frailty using the Clinical Frailty Score. Information on return to work, pre- and post-morbid activity/exercise levels and prevalence of falls was also collected through direct interviews with participants using standardised subjective assessments.

#### 6-Minute walk test

The 6MWT is a sub-maximal exercise test used to assess aerobic capacity and endurance [16], commonly used as a one-time measure of functional status of patients with multiple chronic diseases [17–21]. In patients with COPD, the intra-rater (Intraclass correlation coefficient (ICC) of 0.98) and inter-rater (ICC of 0.96) reliability of the test are strong [22] and the test is highly reliable for assessing exercise capacity in patients with congestive heart failure over-time (ICC = 0.96) [23].

Heart rate, oxygen saturations and the Modified Borg Dyspnoea Scale (MBS) were monitored throughout the test. The MBS (range 0–10) has been widely used to assess perceived exertion in both healthy and diseased states [15, 24].

#### Clinical Frailty Score

Frailty was assessed using the Clinical Frailty Score (CFS) [25]. The CFS is a clinician judgement-based frailty tool that evaluates specific domains including comorbidity, function, and cognition to generate a frailty score ranging from 1 (very fit) to 9 (terminally ill) [26]. The tool’s inter-rater reliability is strong (weighted kappa 0.86) [27].

### Subjective measurements

Patient reported outcomes examining fatigue, quality of life and patient concerns were assessed using standard questionnaires.

#### Chalder Fatigue Scale

Fatigue was assessed using the validated Chalder Fatigue Scale, a self-administered, 11-item questionnaire [28]. At each timepoint participants compared their fatigue during the past month to pre-COVID-19 baseline. Each of the 11 items are answered on a 4-point scale ranging from the asymptomatic to maximum symptomology. Participants can score between 0 and 33 spanning two dimensions (physical and psychological fatigue) with higher scores suggesting worse fatigue. The tool closely resembles other fatigue questionnaires [28–30].

#### Short-form 36 Health Survey Questionnaire (SF-36-II)

Health-related quality of life (HrQOL) was assessed using the Short-form 36 Questionnaire Version 2 (SF-36-II). It covers eight health domains: physical functioning (PF), bodily pain (BP), role limitations due to physical health problems (RP), role limitations due to personal or emotional problems (RE), emotional well-being (MH), social functioning (SF), energy/fatigue (VT), and general health perceptions (GH). Scores for each domain range from 0 to 100, with a higher score indicating a more favorable health state [31].

#### Covid-19 specific patient concerns assessment

The Patient Concerns Assessment tool used in this study was adapted from the National Comprehensive Cancer Network Clinical Practice Guidelines in Oncology for Distress Management (V.2.2013) [32]. It compromises a list of concerns or symptoms which participants can ‘tick’ if these are still bothersome at each time-point during their recovery. The frequency of each symptom at each time-point was calculated.

The assessment battery was repeated at T2 and T3.

### Statistical analysis

Statistical analysis was carried out using STATA v15.0 (Texas, USA). Data visualisation was performed using GraphPad Prism v9.0 (California, USA). Descriptive statistics are reported as means with standard deviations (SD) and median with interquartile ranges (IQR) following Shapiro-Wilks testing for normality. Univariate analysis was performed using t-test, Wilcoxon rank-sum, and Chi-squared test as appropriate. Paired analysis between matched samples from the same patients were carried out using one-way analysis of variance (ANOVA) with post-hoc Tukey testing as appropriate. Comparison of SF-36 scores with normative data was performed using Kruskal–Wallis testing with Dunn’s multiple comparisons test (Additional file 2: Fig. 4). Multivariable linear regression is reported as beta-coefficients with corresponding 95% confidence intervals and *p* values. Variables included in regression analyses are stated in the relevant results section and table legends (Additional file 1: Tables S3 and Table S4). Correlation analysis between parameters was performed using Spearman correlation tests. Specific tests used and adjusted significance levels are stated in the relevant figure/table legends. Statistical significance was considered *p* < 0.05.

## Results

**Fig. 1:**
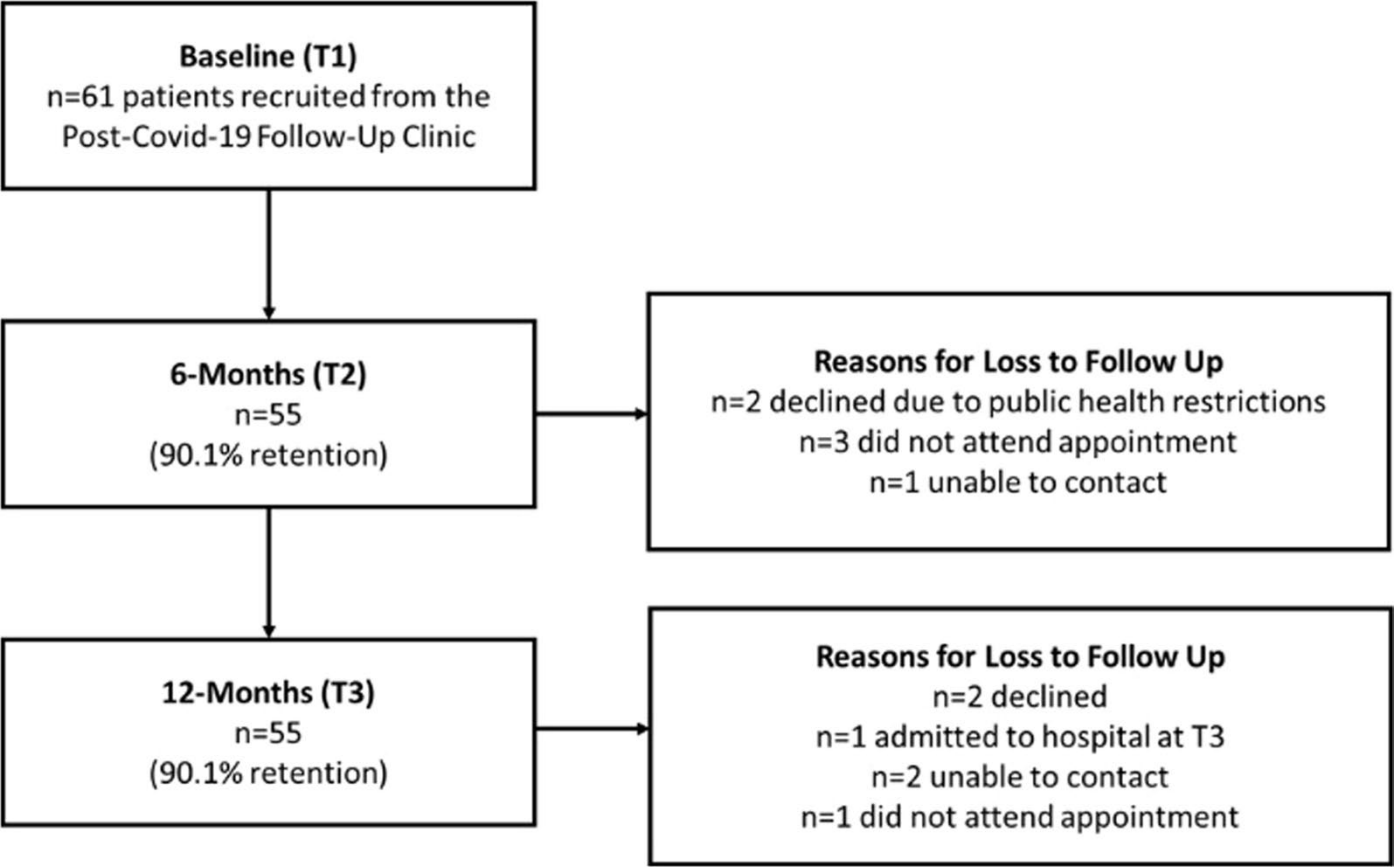
Participant flowchart. Of 61 participants enrolled in the study, all 61 were assessed at least once. Three (4.91%) were assessed only once (all at 3-months post hospital discharge). Seven (11.45%) patients were assessed twice only (n = 3 at 3 and 6-months, n = 1 at 6 and 12-months and n = 3 at 3 and 12-months) and the remaining 51 (83.6%) patients were assessed 3 times

Between May and November 2020, 61 patients were referred to physiotherapy and consented to take part in this study (Fig. 1). Assessments were conducted at three timepoints; Timepoint 1 (T1) at median 55 days (IQR 41.26–63) post-discharge, Timepoint 2 (T2) at median 242 days (IQR 219.75–261.25) post-discharge, and Timepoint 3 (T3) at median 430 days (IQR 398–458) following discharge. Demographics for the study cohort can be found in Table 1.

**Table 1:** Patient demographics for the study cohort

Demographic	n (%)
Gender	
Male—n (%)	35 (57)
Female—n (%)	26 (43)
Age—*years (mean, SD)*	58.6 (13.1)
Occupation	
Retired pre COVID-19—*n (%)*	22 (36)
Unemployed pre COVID-19—*n (%)*	6 (9.8)
Working pre COVID-19—*n (%)*	33 (54.1)
Healthcare workers—*n (%)*	17 (27.9)
Co-morbidities—no. *(median, IRQ)*	1 (0–3)
Length of stay—*days (median, IRQ)*	13.1 (5.8—18.1)
Acute infection	
Requiring admission to ICU—*n (%)*	16 (26)
Requiring mechanical ventilation—*n (%)*	10 (16)
Requiring supplemental oxygen—*no (%)*	42 (69)
Received inpatient physiotherapy—*no (%)*	35 (57)
Received steroid therapy—*no (%)*	19 (32)
Total	61

### Objective measures of recovery

Physical recovery was assessed at all three timepoints using the 6MWT, return to work, prevalence of falls and the Clinical Frailty Score. Return to pre-COVID-19 exercise levels was also assessed at T2 and T3. The median 6MWD covered at each timepoint is shown in *Table **2*. There was a significant improvement in distance covered across these timepoints (*F* = 10.3, *p* < 0.001) and this improvement was independent of illness severity, age and sex. Despite the improvement in distance covered, there was no significant change in perceived exertion, as assessed by MBS (Table 2). Mean 6MWD were considerably less than that of population norms (e.g. average 6MWD at T3 was 447 m compared to population norms of 572 m for males and 538 m for females) [33].

**Table 2:** Physical functioning outcomes of hospitalised COVID-19 survivors

Outcome	Timepoint 1 (N = 60)	Timepoint 2 (N = 55)	Timepoint 3 (N = 55)	p value	F value
Objective measures of recovery					
6MWTD *(mean, SD)*	365 (209)	421 (92)	447 (85)	0.0001	10.13
MBS *(mean, SD)*	3.5 (2.4)	3.2 (2.6)	2.5 (2.4)	0.15	1.93
Return to work n = 33					
Full employment, *n (%)*	13 (39)	15 (45)	18 (55)	0.32	1.17
Reduced hours, *n (%)*	1 (3)	3 (9)	4 (12)		
No return to work, *n (%)*	19 (58)	15 (46)	11 (33)		
Exercise (N = 53)					
Same exercise levels pre- and post-COVID-19, *n (%)*	N/A	24 (45)	27 (51)	0.78	0.08
Lower exercise levels pre- and post-COVID-19, *n (%)*	N/A	29 (54)	26 (49)		
Falls					
Participants reporting falls following hospital discharge, *n (%)*	5 (8) (between DC and T1)	4 (7) (between T1 and t = T2)	0 (between T2 and T3)		
Frailty					
Clinical Frailty Score *(median)*	3	2	2		
Subjective measures of recovery					
Fatigue					
Chalder Fatigue Score *(mean, SD)*	17.5 (6.5)	16.7 (5.9)	16.7 (5.6)	0.73	0.32
Health-Related Quality of Life, Short-Form 36 Scores					
PF *(mean, SD)*	62 (24)	61 (25)	64 (25)	0.85	0.17
rp *(mean, SD)*	39 (39)	50 (43)	54 (45)	0.20	1.65
RE *(mean, SD)*	58 (43)	64 (41)	65 (44)	0.70	0.36
VT *(mean, SD)*	47 (24)	52 (22)	49 (21)	0.50	0.70
MH *(mean, SD)*	70 (23)	74 (18)	73 (19)	0.60	0.52
SF *(mean, SD)*	66 (29)	76 (26)	77 (26)	0.10	2.29
BP *(mean, sd)*	65 (30)	65 (27)	68 (26)	0.82	0.20
GH *(mean, SD)*	61 (20)	55 (19)	54 (19)	020	1.65
Health change *(mean, sd)*	44 (28)	40 (25)	61 (31)	0.0005	8.04
Patient Concerns					
Number of concerns reported *(median, IRQ)*	4 (7)	3 (7)	4 (7)		
Participants reporting fatigue, *n (%)*	24 (40)	27 (49)	21 (38)		
Participants reporting issues with memory/concentration, *n (%)*	22 (37)	19 (35)	27 (49)		

Unknown or missing data: Chalder Fatigue Score: 0 (10-weeks), 1 (6-months) and 1 (12-months). 6MWTD: 0 (10-weeks), 4 (6-months) and 3 (12-months). SF-36-II: 9 (10-weeks), 2 (6-months) and 1 (12-months). Return to work: 0 (10-weeks), 0 (6-months) and 0 (12-months). Patient concerns/symptoms: 0 (10-weeks), 0 (6-months) and 1 (12-months). Exercise levels: N/A at 10-weeks, 0 (6-months) and 0 (12-months). Falls: 0 (10-weeks), 0 (6-months) and 0 (12-months)

*SD* standard deviation, *N/A* not assessed, *IQR* interquartile range, *HrQOL* Health-related quality of life, *PF* physical function, *RP* role limitations due to physical problems, *RE* role limitations due to emotional problems, *VT* energy/vitality, *MH* emotional well-being, *SF* social functioning, *BP* bodily pain, *GH* general health

p values are calculated for comparisons across the 3-month, 6-month and 12-month assessments using linear mixed models for continuous variables or cumulative link mixed models for ordinal variables

Thirty-three participants had been employed prior to their hospitalization. Of those, 13 (39%), 15 (45%) and 18 (55%) returned to work full-time at T1, T2 and T3, respectively. Of the 17 health-care workers enrolled, 71% had returned to work fully, 11% had returned on reduced hours, while 18% had not returned at all at 1-year.

Prior to hospitalization with COVID-19, 53 (87%) of participants had undertaken regular exercise. There was no difference between return to exercise at T2 and T3 (*n* = 24 and *n* = 27, *F* = 0.08, *p* = 0.78; Table 2). Those who had returned to pre-COVID-19 exercise levels at T3 had significantly lower breathlessness scores during the 6MWT (p = 0.02) than those who had not.

At T1, 5 (8.3%) participants reported having at least one fall since their discharge from hospital, with a further 4 (7.27%) reporting falls at T2. No further falls were reported at T3. There was a significant association between the prevalence of falls and increasing frailty scores (p = 0.002) (R^2^ = 0–39). The median frailty scores were 3, 2 and 2 at T1, T2 and T3 respectively.

### Subjective measures of recovery

**Fig. 2:**
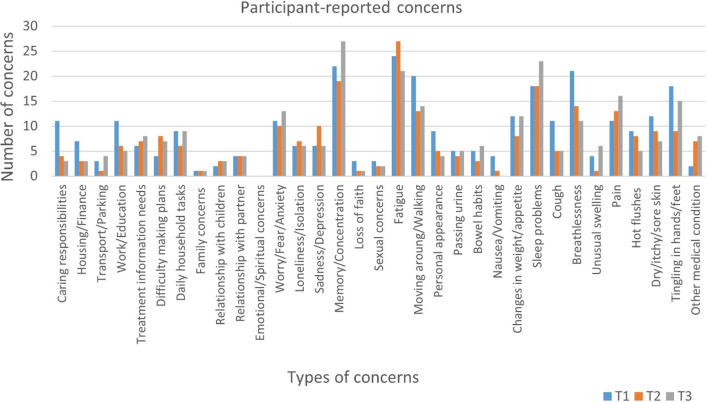
A breakdown of participant concerns across 3 timepoints

Participants were asked to identify a range of COVID-19-related concerns. At least one concern was reported by 44 (74%), 32 (59%) and 35 (64%) participants at T1, T2 and T3 respectively, while the median number of concerns reported per participant across timepoints were 4, 3 and 4 (Table 2). Fatigue was the most frequently reported symptom at T1, n = 24 (40%) and T2, n = 27 (49%), while issues with memory/concentration was the most frequently reported at T3, n = 27 (49%). Breathlessness was reported by 23 (38%), 14 (25%) and 11 (20%) participants at T1, T2 and T3 respectively. A further breakdown of participant-reported concerns can be seen in Fig. 2.

Fatigue was formally assessed using the Chalder Fatigue Score. The median fatigue scores are shown in Table 2. Twenty-eight (46%) participants met the case definition for fatigue at T1, with 29 (48%) meeting it at 2 and 26 (43%) at T3. Fatigue scores for the cohort as a whole did not significantly differ across timepoints (Table 2). However, of the 28 participants who met the case definition for fatigue at T1, fatigue scores demonstrated significant improvement at both T2 and T3 (χ^2^ 12.88, p < 0.01). Those who had returned to self-reported pre-COVID-19 exercise levels had significantly lower fatigue scores at time-point 2 (p = 0.0249). At T1, T2 and T3, 18 (30%), 18 (33%) and 23 (42%) participants reported new sleep problems since infection with COVID-19.

A broader quality of life assessment was performed using the SF-36. There was no significant difference in mean scores of the eight domains of HrQOL over the 1-year period, however, there was significant improvement in the single-item health-change score (p = 0.0005, *F* = 8.04). Improvement in this score were observed between T2 (when participants compared their general health to pre-COVID-19) and T3 (when participants compared their general health to the time of their acute infection with COVID or just after). Nonetheless, 30 (55%) participants at T2, and at 17 (31%) at T3, felt their general health was worse than 1-year prior. To further contextualise the SF-36 results, they were compared to normative population data. Post-COVID-19 patients demonstrated significantly lower scores in physical functioning and energy/vitality at all three timepoints, as well as role limitations due to physical problems at T1 and T2 and general health domains at T2 and T3 (Additional file 2: Fig. S4) [34].

**Fig. 3:**
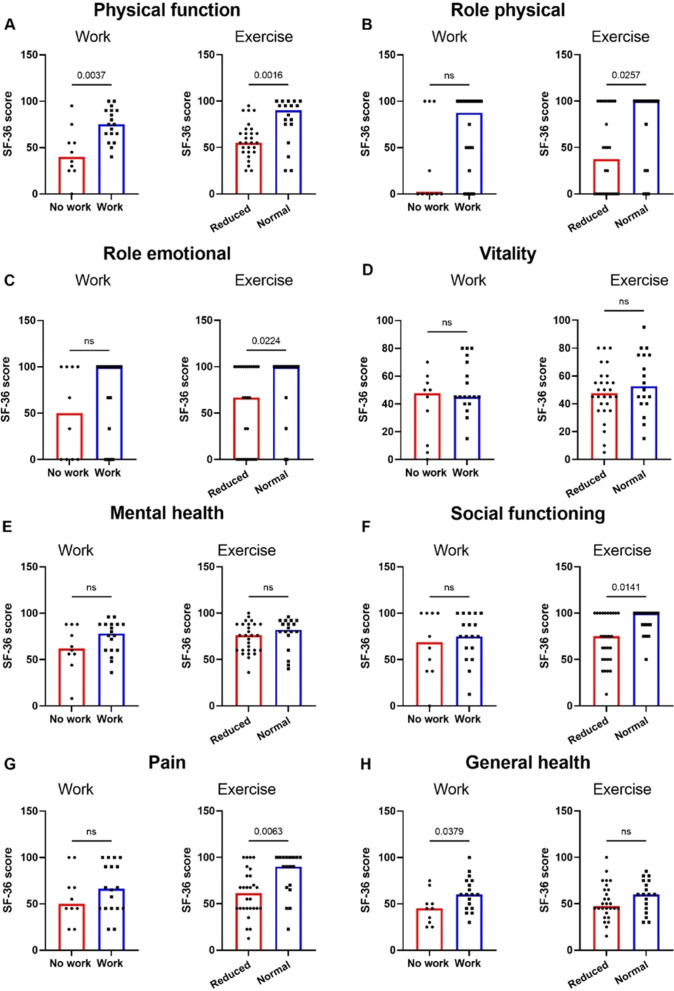
Comparison of those returned to work and those returned to exercise and those who have not across the SF-36 domains **A** Physical function, **B** Role physical, **C** Role emotional, **D** Vitality, **E** Mental health, **F** Social functioning, **G** Pain, and **H** General health. Differences assessed using Wilcoxon rank-sum. *ns* not significant

Finally, the associations between failure to return to functional baseline and subjective measures of health were investigated. Scores across the 8 SF-36 domains were compared between those who had returned to work (n = 18) and those who had not (n = 15), as well as those who had returned to pre-COVID-19 exercise levels (n = 27) and those who had not (n = 26), at T3. Participants who had not returned to employment at 1-year reported worse physical function (PF) and general health (GH) measures. Those who had not returned to pre-COVID-19 exercise levels also demonstrated worse physical function (PF), as well as significantly lower scores on physical and emotional impact on their life (RP and RE respectively), worse social functioning (SF), and worse pain scores (Fig. *3*). These results remained significant when adjusted for sex, age and severity of initial infection (Additional file 1: Table S3, Table S4).

## Discussion

We report on objective and subjective markers of physical recovery in a prospective, hospitalised, COVID-19 cohort followed over 12-months following hospital-discharge. Overall, while participants experienced some recovery as demonstrated by improvements in 6MWD, many report ongoing symptoms. When compared to population norms, HrQOL amongst our cohort is lower, with fatigue, breathlessness and issues with memory/concentration persisting long after hospital-discharge. Return to work rates are low amongst our cohort, with just over half of patients returning to work in some capacity, and a substantial percentage of participants don’t feel back to full-health at 1-year following infection.

We are aware of only four other published studies looking at 1-year outcomes of COVID-19 survivors [35–38]. One of these studies focuses on recovery amongst those who experienced COVID-19-associated Acute Respiratory Distress Syndrome (ARDS) [36], while two studies concentrate solely on respiratory outcomes [37, 38].

In contrast to findings of an Italian cohort of COVID-19 associated ARDS survivors [36], we observed a significant improvement in mean 6MWD over time, with the largest improvement occurring between 10 weeks and 6-months. A small percentage of our patients experienced COVID-19 related ARDs and the reason for this improvement may be multifactorial. Breathlessness was reported less frequently in our cohort as time progresses and there was a slight decrease in MBS breathlessness scores during the 6MWT. Patients also walked at a faster pace, covering a greater distance in the 6-min. As most activities of daily living are carried out at a submaximal intensity, this improvement may be felt by patients when carrying out housework, walking, climbing stairs etc. In contrast, Huang and colleagues found that breathlessness of ≥ 1 on the Modified Medical Research Council Dyspnoea Scale were more prevalent in their study cohort between 6 and 12-months and this was found in conjunction with no improvement in 6MWD between these two time-points [35]. Those who had returned to their pre-COVID-19 levels of exercise were less breathless during 6MWT, this then poses the question, does participating in physical activity aid recovery and possibly reduce persistent symptoms, or simply do those who experience less breathlessness following COVID-19 feel more able to return to exercise.

The proportion of patients who were substantially fatigued at T2 and T3 was much higher than that observed in the general population (9.7%) [39]. Fatigue was the most reported symptom/concern at T1 (49%) and T2 (38%). At T3, 34% reported persistent fatigue. In a healthy population, fatigue is associated with perceived stress and self-perceived health status [39]. When one considers the traumatic experience COVID-19 patients faced when hospitalised during a pandemic with an unknown novel virus, it is not surprising that many patients experience fatigue during recovery. As 27% of SARS survivors met the criteria for chronic fatigue syndrome 4 years following acute illness [40], it is important that COVID-19 survivors are followed up over the same prolonged timescale to better understand the trajectory and recovery of fatigue. It is possible that the fatigue experienced by some participants in our cohort may be attributable to the sleep problems patients are reporting. Interestingly, at 6-months, patients who had returned to pre-COVID-19 exercise levels had significantly lower fatigue scores. It is difficult to know whether patients who were less fatigued felt more able to exercise or if exercise helps to alleviate post-COVID-19 fatigue, this is something which should be explored in future research.

Many patients reported having at least 1 concern or symptom at all study timepoints (72%, 56% and 60% at T1, T2 and T3 respectively). In contrast, Huang and colleagues reported a significant decrease in self-reported persistent symptoms from 6-months to 12-months (68% at 6-months and 49% at 12-months) [35]. We observed an increase in self-reported worry/fear/anxiety between 6-months and 1-year, consistent with Huang and colleagues. At 1-year there was also an increase in the number of participants reporting issues with memory and/or concentration. The mechanism underpinning these symptoms is not entirely understood. However, it is important that indirect effects of the COVID-19 pandemic are considered too, including recovering from hospitalisation during a lockdown, reduced social contact, inability to return to work due to ongoing illness or government mandated restrictions and living in a period of uncertainty to name just a few. Further qualitative research is warranted to investigate patients’ perceptions of their recovery from a severe illness during a global pandemic.

The return-to-work rates amongst our cohort is considerably lower than that observed in other studies of hospitalised COVID-19 patients which report that up to 88% of participants return to work at 12-months [35, 36]. Of those who had not returned to work at 1-year, 70% reported persistent issues with memory and concentration at this time, which along with physical limitations must contribute to work absenteeism. Of the 17 health-care workers in our study, 71% had returned to work fully, 11% had returned partially, while 18% had not returned at all. With an already under-pressure health-care system, with many of its employees contracting COVID-19 in the workplace, having just over 70% of these employees returning to previous levels of work is not ideal. As many health-care workers were infected in the workplace, this brings its own set of challenges and complexities to their recovery and subsequently their return to work. Appropriate follow-up and vocational rehabilitative strategies should be prioritised in supporting individuals’ return-to-work.

Participants in this study demonstrated significantly lower average scores in the PF, RP, VT and GH domains of the SF-36, when compared to normative data [34], similar to trends observed in SARS survivors at 12-months [13]. Although there was a significant difference in health change (p = 0.0005), between T2 and T3, 31% of patients felt their general health was worse 1-year after infection with COVID-19. It is noteworthy, that there were significant differences in HrQOL scores between those who had returned to both work and exercise at 1-year following hospitalization compared to those who had not. These findings solidify the need for appropriate follow-up for those recovering from COVID-19, to offer appropriate rehabilitative services required to support return to functional baseline.

### Limitations

Despite this study being conducted during a global pandemic, with government restrictions in place throughout the study period, retention rates for follow-up at both timepoints were high. However, our sample size is relatively modest. Moreover, we do not have pre-infection physical functioning data. There may also be some selection bias as participants were referred to physiotherapy at the Post-COVID-19 clinic. It is also worth noting that this study was conducted during the 1^st^ wave of the COVID-19 pandemic when management and treatment of these patients were being determined in real-time. This had implications in decision-making surrounding who should be hospitalised. The acuity of hospitalised patients may be higher in subsequent waves and therefore have more complex recovery.

## Conclusion

One-year following hospitalisation with COVID-19, survivors in this study are still reporting persistent symptoms including, fatigue, breathlessness, sleep problems, issues with memory and concentration and more. These persistent symptoms are affecting their ability to return to previous levels of function. This impairment in function has resulted in reduced quality of life. Further research is warranted to explore the effects of various interventions such as exercise therapy and other rehabilitative strategies on patient outcomes such as exercise tolerance, fatigue levels and persistent symptoms in those recovering from COVID-19.

## Supplementary Information

**Additional file 1:** The effects of severity of initial infection, as well as age and sex, may also affect return to work and exercise post-COVID-19. A multiple linear regression model was built for the seven SF-36 parameters that demonstrated significant differences on univariate testing, with the addition of sex, age and severity of initial infection. Severity was based on the WHO severity grading system. Following these adjustments, all significant results on univariate testing remained significant (Table 3, Table 4).

**Additional file 2: Figure 3.** Results across 8 SF-36 domains compared with normative population data {Jenkinson, 1993 #47} at T1, T2 and T3. Kruskal-Wallis test with post-hoc Dunn’s multiple comparison test used to assess differences. ns=not significant.

## Data Availability

Datasets used and analysed are available from the corresponding author upon reasonable request.
