# Supplementary material for: 1-year quality of life and health-outcomes in patients hospitalised with COVID-19: a longitudinal cohort study
Source: Respir Res. 2022 May 4;23:115. doi: 10.1186/s12931-022-02032-7 (PMC9067558; doi:10.1186/s12931-022-02032-7)
Supplement: Supplementary file 2 — Additional file 2: Figure 3. Results across 8 SF-36 domains compared with normative population data {Jenkinson, 1993 #47} at T1, T2 and T3. Kruskal-Wallis test with post-hoc Dunn’s multiple comparison test used to assess differences. ns=not significant. [file 12931_2022_2032_MOESM2_ESM.docx]

The effects of severity of initial infection, as well as age and sex, may also affect return to work and exercise post-COVID-19. A multiple linear regression model was built for the seven SF-36 parameters that demonstrated significant differences on univariate testing, with the addition of sex, age and severity of initial infection. Severity was based on the WHO severity grading system. Following these adjustments, all significant results on univariate testing remained significant (*Table 3, Table 4*).

Table 3

Table 4:

|  | Physical function | | Role physical | | Role emotional | | Social function | | Pain | |
| --- | --- | --- | --- | --- | --- | --- | --- | --- | --- | --- |
|  | β coefficient (95% CI) | *P* value | β coefficient (95% CI) | *P* value | β coefficient (95% CI) | *P*  value | β coefficient (95% CI) | *P*  value | β coefficient (95% CI) | *P*  value |
| Exercise | -22 (-37--8) | 0.01 | -35 (-63- -6) | 0.02 | -28 (-56 – -1) | 0.04 | -20 (-35 – -6) | 0.01 | -23 (-39 – -7) | 0.01 |
| Severity | -1 (-7 – 6) | 0.85 | -4 (-18 – 9) | 0.55 | 2 (-11 – 16) | 0.71 | 0.2 (-7 – 7) | 0.96 | -3 (-11 – 5) | 0.46 |
| Age | -0.2 (-0.7-0.3) | 0.44 | 1 (-13 – 44) | 0.61 | 0.4 (-0.6 - 1.4) | 0.42 | 0.3 (-0.3 – 0.8) | 0.33 | -0.2 (-0.8 – 0.4) | 0.58 |
| Sex, female | 6 (-9 – 20) | 0.44 | 16 (-13– 45) | 0.28 | 45 (-49 – 139) | 0.34 | 8 (-7 – 23) | 0.29 | 1 (-16 – 17) | 0.91 |
